# UNDERSTANDING COMMUNITY RESOURCES FOR OLDER ADULTS WITH DISABILITIES

**DOI:** 10.1093/geroni/igae098.3916

**Published:** 2024-12-31

**Authors:** Sunghei Han, Sojung Park, Soobin Park, Esther Shin, Sarah Kang, Eunsun Kwon, Seoyeon Ahn, BoRin Kim

**Affiliations:** Washington University in St. Louis, St. Louis, Missouri, United States; Washington University in St. Louis, St. Louis, Missouri, United States; Washington University in St. Louis, St. Louis, Missouri, United States; Illinois State University, Normal, Illinois, United States; University of California Los Angeles, Los Angeles, California, United States; Fairleigh Dickinson University, Hackensack, New Jersey, United States; Duke-NUS Medical School, Singapore, Singapore; University of New Hampshire, Durham, New Hampshire, United States

## Abstract

For older adults with various disabilities—physical, mental, cognitive, and others—diverse community resources are essential for daily functioning and their Aging in Place (AIP). This scoping review analyzed 21 peer-reviewed studies from databases like PsycINFO, CINAHL, MEDLINE, Scopus, and PubMed to identify gaps in research on community resources for this population. Thematic analysis revealed four main themes: 1) the development of partnerships among local governments, service providers, and stakeholders to coordinate resources; 2) evaluation of community-level interventions such as telerehabilitation and mental health programs; 3) factors affecting access to and use of community resources; and 4) the impact of these resources on health and well-being. The studies primarily focused on home and community-based services (HCBS), but while some addressed specific care aspects like fall prevention and dementia care, broader research often overlooked the comprehensive needs of older adults with disabilities. Only two studies focused on resources for mental health and substance use disorders, highlighting significant gaps, particularly in primary care. Additionally, many studies lacked clear definitions of disabilities, leading to a fragmented understanding of community resources. This lack of clarity hinders efforts to address the unmet needs of older adults with disabilities, including diverse subgroups like LGBTQ individuals and ethnic minorities. The study highlights the need for more systematic research into the availability and accessibility of community resources to support older adults with varying disabilities better, ensuring no critical needs are left unmet in this vulnerable population.

